# Disclosure to HIV-seropositive children in rural Zambia

**DOI:** 10.1186/s12887-018-1252-2

**Published:** 2018-08-18

**Authors:** Shinya Tsuzuki, Naoko Ishikawa, Hideki Miyamoto, Christopher Dube, Nangana Kayama, Janet Watala, Albert Mwango

**Affiliations:** 1 National Center for Global Health and Medicine, 1-21-1 Toyama, Shinjuku-ku, Tokyo, 162-8655 Japan; 2 Mumbwa District Community Health Office, Mumbwa, Zambia; 3 Ministry of Health Zambia - JICA SHIMA Project, Lusaka, Zambia; 4 Ministry of Health Zambia, Lusaka, Zambia

**Keywords:** HIV, Children, Disclosure, Zambia

## Abstract

**Background:**

Care of children living with HIV comprises various issues, some considered challenging. One of the challenging areas is the serostatus disclosure to HIV-positive children. This study describes the current situation of HIV disclosure among rural children in Zambia and examines the socio-demographic factors promoting disclosure.

**Methods:**

We used a mixed method approach applying both quantitative and qualitative methods to obtain comprehensive picture of HIV serostatus disclosure for children. Data were collected in Mumbwa district, Zambia (2010–2012), included 57 clinical records of children older than 5 years old. We examined children’s age, gender, and cohabitation status with their parents, caregivers’ level of education and income, and the relation between children and caregivers. Logistic regression model was applied to examine associations between disclosure and socio-demographic characteristics. Semi-structured interviews with 50 caregivers and 22 HIV-positive children were conducted to qualitatively investigate attitude towards disclosure and support needed.

**Results:**

Full disclosure was completed in 17 out of 57 (29.8%) patients. Median ages of patients in disclosed group and non-disclosed group were 10 and 9, respectively (IQR 8.0–13.0, 7.0–11.25). In univariate analyses, older age and male gender has positive relation to the completion of serostatus disclosure. In logistic regression models, cohabitation status with patients’ mothers showed positive correlation to the completion of serostatus disclosure. In the interviews with caregivers, all caregivers said that disclosure of serostatus is a necessary process and good for their children, while actual serostatus disclosure rate was low.

**Conclusion:**

Serostatus disclosure to HIV-seropositive children is not prevalent in Rural Zambia. Although further researches would be desirable, increased support to caregivers would be beneficial.

**Electronic supplementary material:**

The online version of this article (10.1186/s12887-018-1252-2) contains supplementary material, which is available to authorized users.

## Background

In 2015 an estimated 1.8 million children under the age of 15 years were living with HIV and about 150,000 children were newly infected with HIV [1]. This number means that 0.4% of children in Africa are living with HIV [2]. Less than one-half of the 2.1 million children age 0–14 years were receiving antiretroviral therapy (ART) [3].

HIV treatment and care for children involves various challenges including disclosure of HIV status [4]. It has gradually been recognized that the disclosure of serostatus can have a positive impact [5–8] and the number of studies of HIV serostatus disclosure to children in resource-limited settings is increasing [9]. Although some previous studies conducted in rural areas of resource-limited settings [10, 11], quantitative evidences are still insufficient.

WHO recommends disclosure of HIV status to HIV-seropositive children aged 6–12 years if they are mature enough to understand the disease [12]. In Zambia, it is recognized that mature children are usually able to deal with the realities of HIV. In 2011, the country developed pediatric guidelines for HIV testing and counselling that included a section on HIV disclosure [13]. However, to date only few studies have examined the implementation of and factors associated with serostatus disclosure in Zambia [10, 14].

Therefore, this study aimed to explore the current situation of HIV serostatus disclosure among children living with HIV in a rural district in Zambia. It examines factors associated with disclosure in children and describes caregivers’ perspectives and behaviors regarding serostatus disclosure.

## Methods

### Data and sample

We used a mixed method approach applying both quantitative and qualitative methods to obtain comprehensive picture of HIV serostatus disclosure for children. We intended to examine factors which promote or prohibit disclosure quantitatively by using clinical data, and at the same time qualitatively investigate attitude towards disclosure and support needed through the interviews.

We reviewed 57 out of 193 patients who were registered at ART clinic in Mumbwa District Hospital, Zambia from 2005 to 2011. Our inclusion criteria are as below; patients who have confirmed information about completion of serostatus disclosure and are ages between 6 and 15. We included patients in accordance with the age at the time of registration, then therefore some patients were over 15 years old at the time of data analysis. We excluded patients who moved out to other districts between 2005 and 2011 (Transout), and who died before full serostatus disclosure had not completed (Death). We also excluded patients whom we lost to follow up at the time of 12 months have passed after registration (LTFU), and who have been followed up less than one year at the end of year 2011 (Short follow-up period). In the present study, we mean “full disclosure” (which means that children know they have a serious illness and it was HIV) when we use the term “disclosure”.

As for interview to caregivers, trained local interviewers conducted semi-structured open-ended interviews. Caregivers attended the clinic between November 2010 and March 2012 were invited for the interview and 50 caregivers agreed to participate in our study. A mobile token was given to caregivers who participated in the interview as a reward. The interviews were conducted in English or in the local languages, Nyanja or Bemba and they lasted approximately one hour. The interviews were recorded with the permission of participants, transcribed, and translated into English. The interviewers collected demographic data of participants and asked caregivers about: trigger of HIV diagnosis, HIV serostatus disclosure, attitudes toward disclosure, and plan for disclosure. We did not exclude caregivers who have children younger than 6 because one of our aim is to understand their attitude towards future plans of serostatus disclosure and their explanation given to children on medication.

Interview to children was conducted in a similar manner to that to caregivers. When caregivers were invited for the interview, trained interviewers asked their children to join the interview. If children understand the objectives of our study and agreed to participate in by themselves, they were included in the study. Finally, we enrolled 22 HIV seropositive children. Children were first asked to draw a picture of a “healthy person” and an “unhealthy person.” Then they were asked to do a drawing exercise called “communication mapping”, in which a child draws pictures of important persons in life and the child’s relationships to them [15]. The drawings were used to initiate the interview with the child. Children were asked about their health condition, their understanding of their disease, and their experience of stigma and discrimination. HIV-related issues were not discussed unless the children voluntarily explained their conditions. For these children who were aware of their HIV-seropositive status were asked about their experience of disclosure.

All the caregivers of children attending the district hospital during the period of November 2010 – March 2012 were invited to the interview and enrollment was ended when their responses seemed to have reached theoretical saturation based on the grounded theory [16]. The details of questionnaire for caregivers and children are available in Additional files 1 and 2, respectively.

Ethical approvals were obtained from the University of Zambia and National Center for Global Health and Medicine. Participation to the study was voluntary and written informed consent was obtained from the caregivers and verbal assent was obtained from the children.

### Analyses

Analysis of quantitative data was conducted using SPSS Statistics for Windows, Version 20.0 (IBM Corp. Released 2011. Armonk, NY: IBM Corp) and R version 3.2.3. Fischer’s Exact test was used for the categorical data; the Mann–Whitney U test was used for the continuous data. We examined children’s age, gender, and cohabitation status with their parents, caregivers’ level of education and income, and the relation between children and caregivers. In the present study, “parents” indicates biological parents of patients, and “caregivers” means persons who take care of patients. Logistic regression analysis was applied to examine associations between disclosure and socio-demographic characteristics. Because we included only 17 children in the disclosed group and 40 children in the non-disclosed group, and 10 cases per one exposure variable is the desired sample number in the smaller outcome group, we used a logistic regression model for adjusting age and at most one other variable [17, 18]. As for choice of variables included in the model, we judged it by external knowledge such as our clinical judgment and literature review [19]. Age was included in all logistic regression models because it is expected that older children are more likely to have had their serostatus disclosed. For all analyses, a *p* value of smaller than .05 was taken to be statistically significant.

For qualitative data, content analysis was conducted in order to obtain in-depth understanding of actual practices and experiences of serostatus disclosure. Transcripts were read line-by-line and coded manually into categories by two members of the research team. Emerging and recurrent themes were sought, findings were discussed among the research team, any discrepancies were reviewed, and consensus was obtained for the final results. Representative quotes covering the range of data were selected to illustrate the themes.

Some discrepancies were found between the clinical records and the results of the interview in five cases. In two cases, the clinical records showed that the children were not aware of their serostatus but the interviewer found that disclosure had already occurred. Conversely, the clinical records showed that three children knew their serostatus but their caregivers said they did not. When a discrepancy was observed, the data from the interviews were prioritized over that from the clinical records because as was often the case with resource-limited settings, clinical records often contain some missing values and/or mistakes due to inadequate preservation system of records. On the other hand, information from interview was obtained by patients’ caregivers who lived with them and mainly take care of their diseases directly, then therefore interview can be regarded as more reliable data source.

## Results

### Quantitative analysis

*Characteristics of children and their caregivers*: 57 clinical records of pediatric HIV care in Mumbwa district hospital were reviewed retrospectively. The median age of the children was 9 years; 28 were male (49.1%) and 29 were female (50.9%). Caregivers’ educational status was reported for 46 caregivers; 17 caregivers (37.0%) had no education or only primary education and 29 caregivers (63.0%) had more than primary education. Caregivers’ monthly income was reported for 42 caregivers; 22 caregivers (52.4%) earn less than 100,000 Zambian Kwacha (1 USD = 5190 ZMW in 2012, before denomination) per month and 20 caregivers (47.6%) earn more than 100,000 ZMW. The main caregiver for children was the mother (43.4%), followed by the grandmother (22.6%).

*HIV serostatus disclosure to children and associated factors*: According to the records, only 17 children (29.8%) were aware of their HIV-seropositive serostatus. Among these, 8 children (47.1%) were aged 11 years or older and 9 children (52.9%) were aged 6 to 10 years. Males were the majority, 12 boys (70.6%) compared with 5 girls (29.4%).

The univariate analyses of factors associated with disclosure showed that children’s age was significantly related to serostatus disclosure (Table 1). The median age of children whose serostatus was disclosed was higher than that of children whose serostatus was not disclosed, according to the result of Mann–Whitney U test (10 years and 9 years, respectively; *p* < .001). The Chi-squared test demonstrated that male children were disclosed more frequently than female children (*p* = .045). Children living with the mother only were more likely to be informed of their HIV status, although it was not statistically significant (*p* = .138). Caregivers’ educational status, and degree of kinship between children and main caregivers were not significantly associated with disclosure. Logistic regression analyses demonstrated that children’s cohabitation status with mother only was significantly associated with disclosure (*p* = .038,) (Table 2). Caregiver’s education level, income, and cohabitation status with father only were not related to disclosure. HIV serostatus disclosure was more common for male children than female children, but this was not statistically significant (*p* = .079).

**Table 1:** Factors associated with HIV serostatus disclosure to children, univariate analysis, Mumbwa district, Zambia, 2012

Factors	Total	Not disclosed	disclosed	*p*-value
Range of patients’ age (median, IQR^a^)	6–17 (9, 7.0–12.0)	6–16 (9, 7.0–11.25)	6–17 (10, 8.0–13.0)	< 0.001^*1^
Gender (%)	57	40 (70.2)	17 (29.8)	0.045^*2^
Male	28	16 (57.1)	12 (42.9)	
Female	29	24 (82.8)	5 (17.2)	
Caregivers’ education (%)	46	32	14	0.520^*3^
None or primary	17	13 (76.5)	4 (23.5)	
Above primary	29	19 (65.5)	10 (34.5)	
Caregivers’ income per month (%)	42	29	13	0.320^*3^
< 100,000 ZMW^b^	22	17 (77.3)	5 (22.7)	
≧100,000 ZMW	20	12 (60.0)	8 (40.0)	
Cohabitation status (%)	39	28	11	
Both parents	17	13 (76.5)	4 (23.5)	1.000^*3^
Mother only	9	4 (44.4)	5 (55.5)	0.138^*3^
Father only	10	8 (80.0)	2 (20.0)	0.710^*3^
No parent	3	3 (100.0)	0 (0.0)	0.554^*3^
Main caregivers (%)	53	36	17	
Mother	23	13	10	0.435^*3^
Grandmother	12	9	3	0.741^*3^
Others	18	14	4	0.556^*3^

^a^Interquartile range; ^b^values are before denomination

*^1^ *p*-value represents significance probability of Mann–Whitney test about median age between “not disclosed” and “disclosed”

^*2^*p*-value represents significance probability of Fisher’s exact test about gender ratio between “not disclosed” and “disclosed”

^*3^*p*-value represents significance probability of Fisher’s exact test between “not disclosed” and “disclosed”

**Table 2:** Fitness of different logistic regression models which adjust age and other one variable

Variable	Odds ratio	Z	*p* > |Z|	95% CI^a^
Model 1 (*n* = 57)				
Age	1.172	1.515	0.130	0.989–1.403
Model 2 (*n* = 57)				
Age	1.281	1.002	0.316	0.934–1.342
Gender (Male)	3.091	1.775	0.079	1.099–9.324
Model 3 (*n* = 46)				
Age	1.209	1.490	0.136	0.985–1.508
Education	1.517	0.586	0.558	0.482–5.172
Model 4 (*n* = 42)				
Age	1.163	1.023	0.307	0.914–1.500
Income	1.871	0.876	0.381	0.578–6.238
Model 5 (*n* = 39)				
Age	1.166	0.894	0.371	0.879–1.558
Living with father only	0.572	−0.614	0.539	0.107–2.333
Model 6 (*n* = 39)				
Age	1.111	0.575	0.565	0.819–1.510
Living with mother only	5.700	2.076	0.038	1.461–23.915

^a^confidence interval

### Caregiver’s interviews

*Current situation of HIV serostatus disclosure*: Fifty caregivers were interviewed. Their characteristics are shown in Table 3. The majority of caregivers (90.0%) were female; 32 caregivers (64.0%) were the child’s mother, 6 caregivers (12.0%) were the child’s grandmother, and 5 caregivers (10.0%) were the child’s aunt. Caregivers’ age ranged from 16 to 64 years (median: 35 years). Age of their children ranged from 0 to 14 years (median: 6 years).

**Table 3:** Demographic characteristics of caregivers interviewed (*n* = 50) and their children, Mumbwa district, Zambia, 2012

Range of caregivers’ age (median, IQR^a^)	Number (%)
16–64 (35, 29–40)	
Gender	*n* = 50
Female	45 (90.0)
Male	5 (10.0)
Relationship to the child	*n* = 50
Mother	32 (64.0)
Grandmother	6 (12.0)
Aunt	5 (10.0)
Father	4 (8.0)
Sister	2 (4.0)
Brother	1 (2.0)
Disclosure	*n* = 50
Yes	9 (18.0)
No	41 (82.0)
Disclosure led by	*n* = 9
Mother	3 (33.3)
Grandmother	2 (22.2)
Parent + Health worker	2 (22.2)
Accidental disclosure	2 (22.2)
Explanation given to child on medication	*n* = 30
No explanation given	6 (20.0)
For sickness	5 (16.7)
Paracetamol	3 (10.0)
For malaria	2 (6.7)
For tuberculosis	2 (6.7)
For cough	2 (6.7)
Vitamin	2 (6.7)
For anemia	1 (3.3)
For growth	1 (3.3)
For polio	1 (3.3)
Cotorimoxazole	1 (3.3)
For swollen lymph nodes	1 (3.3)
To become clever	1 (3.3)
others	2 (6.7)

^a^Interquartile range

The current situation of HIV serostatus disclosure is also shown in Table 3. Of 50 HIV-seropositive children, only 9 (18.0%) were reported to be aware of their HIV status. In 3 of the 9 cases, disclosure was initiated by mother; in 2 cases, grandmother decided disclosure; and in 2 cases disclosure was led by health workers with family members.

*When he was 6 years old, he asked me why we must take the medicine. I explained him that we were both HIV positive so this was the medication for the disease. He was worried for a while but later became a happy child again. (Mother age 39 years; boy age 9 years)*

*Today the nurse at clinic and myself explained to him. I told him that when he was a baby, he was drinking breast milk from his mother and sometimes bit her breast, so from that he got the disease, that’s why he took medicine every day because (he was) HIV positive. But I also told him that it was not his mother’s fault and that I love him. (Father age 46 years; boy age 12 years).*

In 2 cases, the children accidentally knew their HIV status from peers and a cousin as presented in the quotes below.

*About a year ago, he went to play football and when he came home, he said that his friends told him that he was HIV positive and he was going to die. He asked me if it was true that he was sick and would die, so I explained him that it was not true. I know how sad he was when his friends told him, so I always avoid talking about it. (Mother 24 year-old; boy 7 year-old)*

*When she had an argument with her cousin, the cousin disclosed her HIV status in a harsh way, saying that those drugs she took were for AIDS. I am planning to explain to her properly maybe when she becomes 14 years. (Aunt 30 year-old; girl 11 year-old)*

Caregivers who had not yet informed children about their HIV serostatus were asked how they explained the medication their children were taking (multiple answers were allowed in this question, details of the results are shown in Table 3). Among 30 caregivers who answered the question, 6 caregivers (20.0%) said they gave medication to the child without any explanation; some caregivers gave some tentative explanations. For example, 5 caregivers (16.7%) explained that the drugs were for “sickness”; 3 caregivers (10.0%) said the medication was paracetamol (acetaminophen). Other explanations included drugs for malaria and tuberculosis, vitamin tablets, and cough medicine.

*Caregivers’ perspectives – Positive attitudes towards disclosure and willingness to lead the process*: Caregivers’ attitudes on HIV serostatus disclosure were analyzed (Table 4). All the caregivers said that children should know their HIV status; they regarded disclosure as an important part of the process of dealing with the disease. Their main reasons were: disclosure is necessary for children to take care of themselves; children should understand about their own disease; children should know their HIV status and that there is no reason to hide it; and, children will ask about the disease.

**Table 4:** Caregivers’ attitudes toward HIV serostatus disclosure to children, Mumbwa district, Zambia, 2012

Caregiver’s attitudes	Total (%)
Children should know their status	*n* = 47
Yes	47
No	0
Reason children should know their HIV status	*n* = 46, multiple answers
Take care of oneself	13 (28.3)
Understand their own disease	12 (26.1)
Need to know/no reason to hide	10 (21.7)
One day child will ask	9 (19.6)
Adherence	4 (8.7)
Child’s right	2 (4.3)
Others	4 (8.7)
Disclosure should be led by	*n* = 40
Mother	20 (50.0)
Mother and other family member	7 (17.5)
Health worker	5 (12.5)
Father	2 (5.0)
Grandmother	2 (5.0)
Family	1 (2.5)
Mother and health worker	1 (2.5)
Others	2 (5.0)

Interquartile range

*She needs to know (her status) because it is very important that in case I am away, she can take (medicine) by herself. (Mother 32 year-old; girl 4 year-old)*

*He needs to understand about his condition and medication to enable him live longer. (Grandfather 50 year-old; boy 3 year-old)*

All caregivers of children who were not yet aware of their serostatus said they were planning to disclose in the future and gave the planned age for disclosure. Twenty-three caregivers (59.0%) said that age between 10 to 12 years was suitable for disclosure of serostatus while 8 caregivers (20.5%) said that older than 12 years was better for disclosure because the child would be able to understand at that age. One caregiver said that she would gradually explain to her daughter about the disease beginning at age 3 years.

Of the 40 caregivers for which data were available, 20 caregivers (50.0%) said that the mother should be the one to inform the child about their serostatus; 7 caregivers (17.5%) said that mother and another family member should lead the disclosure; and 5 caregivers (12.5%) said that health workers should inform the children about their serostatus.

### Children’s interviews

**Fig. 1:**
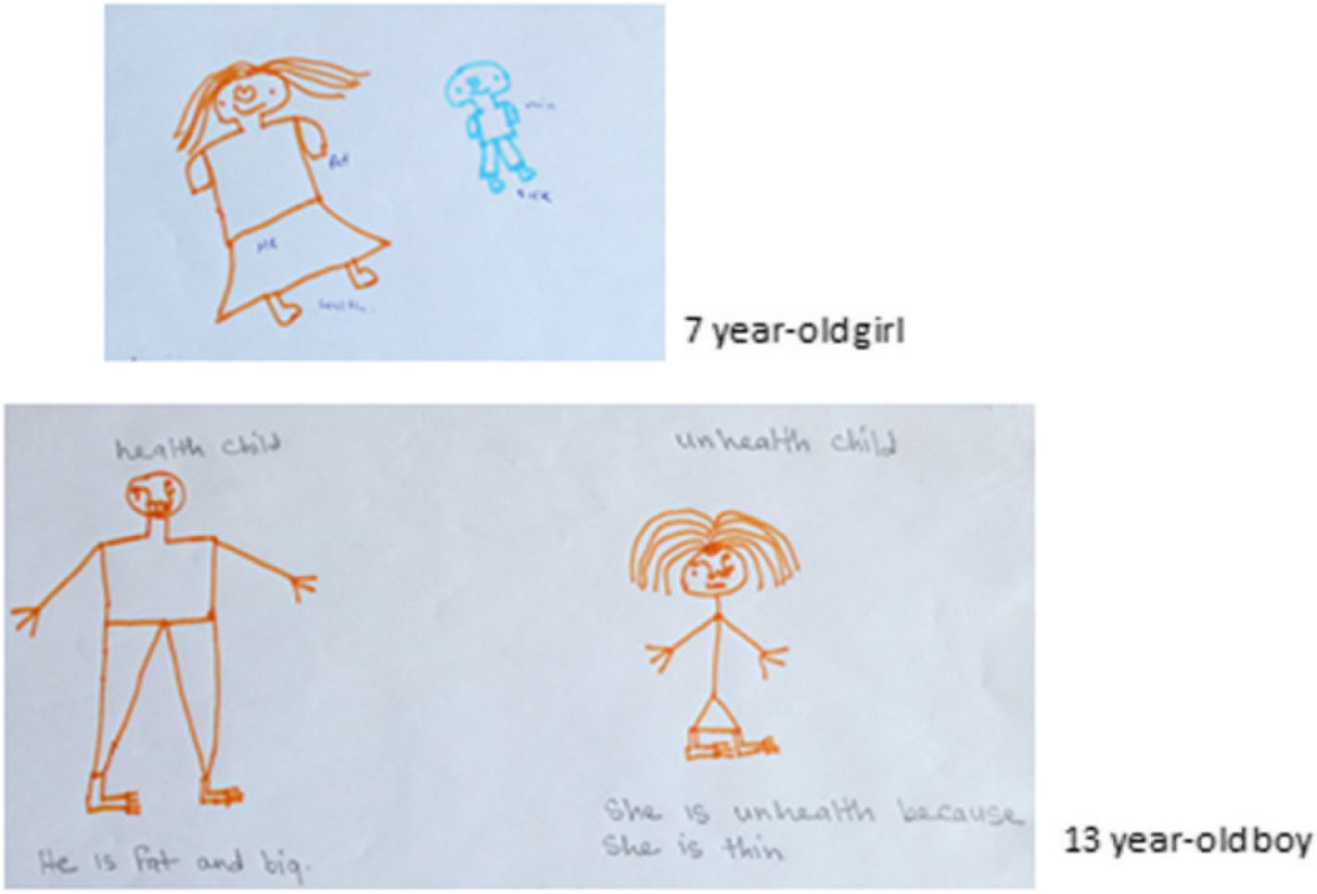
HIV-positive children’s perceptions of “healthy” and “unhealthy” persons, Mumbwa district, Zambia, 2012

Twenty-two children (11 girls and 11 boys, age 5 to 13 years) were interviewed in the study. Figure 1 shows an example of a child’s drawing of a “healthy person” and an “unhealthy person.” Among 22 children, 9 said they were “healthy” and 6 said they were “unhealthy.” Three children said the reason they felt they were unhealthy was because they were thin compared with other children; 5 children said they felt “different” from other children. Overall, 12 of the 22 children said they felt the same as the other children.

*Limited serostatus disclosure*: When asked the reason for the clinic visit (multiple answers were allowed in this question), 14 children said that it was to obtain medication. Three children said that the medication was for tuberculosis; one said it was for malaria; and four children said that it was for other reasons, including cough, chest pain, and headache. Nine children said they did not know for what the medications were for; only three children reported that the medications were antiretroviral drugs. The responses of children and their caregivers matched in two cases.

*Acceptance of own serostatus*: Three of the 22 children (1 girl and 2 boys, age 12–13 years) said they were aware of their HIV-seropositive status.

*My mother told me that I was HIV positive and that I was not the only one and it’s not my fault. I didn’t feel good, I felt sad and cried. (girl age 13 years)*

*My mother told me that I should always take medicine because I’m sick. I was told that when I miss my medication I could die and that I should always follow time. My grandfather encourages me to take the medicine. He says I will grow up and continue taking them by myself when they die. I felt ok. I was very sick and I didn't have strength so it’s ok to take the medicine because now I am ok. (boy age 12 years)*

*My father explained to me that I got disease from mother. I felt happy to know the truth. My father said that when I took this medicine every day, I would be healthy. Health worker explained that if I took my medicine virus will sleep. If I take the medicine, I will be healthy. (boy age 12 years)*

*The other 19 children were not aware of their HIV-seropositive status.*

## Discussion

To our knowledge, this is one of few studies to examine the situation of HIV serostatus disclosure in children and associated factors in a rural district of Zambia. According to the quantitative data, children’s age, gender and parental cohabitation status are related to disclosure. The qualitative data showed positive attitudes of caregivers toward disclosure to children.

In general, HIV disclosure rates for HIV-positive children are low in sub-Saharan Africa [9]. In our study, only 29.8% of seropositive children knew their HIV status, a prevalence rate similar to that in Tanzania and Uganda [20, 21]. Furthermore, the present study demonstrated that there is no outstanding difference about prevalence rate of disclosure between urban and rural areas [9, 22]. Univariate analysis in our study demonstrated that patients’ age and gender are associated with serostatus disclosure. Older children and male children were more frequently informed of their serostatus. It is expected that children’s age is associated with disclosure status because their comprehension grows with age. A systematic review, along with recent work by some researchers, have noted this pattern of age-related disclosure [9, 20, 21], and our results supported the finding. However, the difference in disclosure rates between male and female children has seldom been reported. Further study is warranted on the relationship between gender and disclosure status.

In logistic regression models, cohabitation status was related to disclosure; children living with their mother were more likely to know their serostatus. However, the influence of gender was insignificant in the logistic regression model. Additionally, as shown in the results of the interviews, qualitative analysis did not show a relationship between children’s gender and disclosure. Therefore, while male children tended to have their serostatus disclosed in the univariate analysis, overall the effect of gender on disclosure should be interpreted with caution.

Several studies have reported positive co-relationships between disclosure and absence of biological father [23]. In our study, children living with the mother only were more likely to have their serostatus disclosed after adjusting the influence of children’s age. Absence of the mother did not demonstrate an influence on the disclosure rates for children who live with and without the father. These tendencies are similar to previous studies [9]. This may suggest that the absence of fathers leads to more frequent or effective disclosure events by mothers. Further consideration and research would be desirable to examine the role of fathers in the process of HIV disclosure in children.

The qualitative part of the study found that caregivers’ attitudes toward serostatus disclosure in children is positive. Most caregivers say that disclosure should be done by the child’s family, especially the mother. Their opinions as to the appropriate age for disclosure were similar to the disclosure age recommended by the Zambian guidelines (age 10 years).

As stated above, despite caregivers’ positive attitude toward disclosure, the context often does not lead to actual disclosure in children; various studies have reported on this problem [14, 24]. The need for interventions to support caregivers in the disclosure process has been suggested [24, 25]. The necessity of interventions is compatible with our results which suggest that health care workers can play a critical role in the process of disclosure. In this context, it is important to link caregivers, children living with HIV, and health care workers.

Although more than half of disclosures were led by children’s family members, accidental disclosures by friends and other persons were also reported, which requires further attention. As a previous study pointed out [26], accidental disclosure might be a more stressful event for children than that by their caregivers and/or health care workers, and it would have negative impact on them contrary to the prepared disclosure process.

Also we should take note on some differences between the quantitative and qualitative data; for example, information about patients’ disclosure status differed between the clinic records and the interviews. It might reflect that disclosure is a gradual process and caregivers’ recognition about its completion is sometimes vague. This means that long-term periods’ cooperation between caregivers and healthcare providers, as the previous studies pointed out [27, 28].

One of the strengths of our study is that it contains both quantitative and qualitative results. Additionally, it focuses on a resource-limited setting in rural Zambia. Together these features distinguish it from most other studies in this area. The principal limitations of the study were the small sample size and some missing data on clinical records. In terms of the qualitative aspects of the study, there were some questionnaire deficiencies regarding absence of parents, cohabitation status, and the actual process of disclosure. These deficiencies could be attributed to delicacy included in serostatus disclosure. Since decision and process of disclosure are quite personal matter, we allowed all interviewees not to answer questions they would not like to. Therefore if participants hesitated to answer any questions, interviewers encourage them to go to next question or even end their interview.

As for quality of data, some missing items in clinical records and several discrepancies between clinical data and interview results might have affected the reliability of our analysis to some extent. 

While this study adds to the body of research on HIV serostatus disclosure to children in resource-limited settings, the limitations of the study dictate that care should be taken in interpreting the results. Further research with larger enrollment in ART programs and more detailed history of patients and caregivers would be desirable to improve the situation of HIV serostatus disclosure among children in rural Zambia.

## Conclusions

In the rural, resource-limited setting of our study, serostatus disclosure to HIV-seropositive children is not prevalent. Further promotion of disclosure to children living with HIV is desirable. Caregivers themselves regard HIV serostatus disclosure positively and think it is necessary to the child’s situation. Increased support to caregivers in disclosure of serostatus to children with HIV would be beneficial.

## Additional files

Additional file 1: Interview guide for caregivers. (DOCX 35 kb)

Additional file 2: Interview guide for chidren. (DOCX 22 kb)

## Abbreviations

AIDS: Acquired Immunodeficiency Syndrome
ART: antiretroviral therapy
HIV: Human Immunodeficiency Virus
USD: United States Dollar
WHO: World Health Organization
ZMW: Zambian Kwacha
