# Supplementary material for: Disclosure to HIV-seropositive children in rural Zambia
Source: BMC Pediatr. 2018 Aug 18;18:272. doi: 10.1186/s12887-018-1252-2 (PMC6098826; doi:10.1186/s12887-018-1252-2)
Supplement: Supplementary file 1 — Interview guide for caregivers. (DOCX 35 kb) [file 12887_2018_1252_MOESM1_ESM.docx]

**Additional file 1**

**Interview guide for caregivers**

**Questionnaire for children’s caregivers**

| Date of interview (dd/mm/yyyy) |  | | | |
| --- | --- | --- | --- | --- |
| Name of interviewer |  | | | |
| 1. **Information of the Child**   (information should be obtained from the patient’s file for gray cells) | | | | |
| 1. Child’s ART number |  | | | |
| 2. Name of child |  | | | |
| 3. Child’s birthday (dd/mm/yyyy) |  | | | |
| 4. Age of child |  | | | |
| 5. Sex of child | 1.□ Boy 2.□ Girl | | | |
| 6. Child’s education | 1.□ Nursery/kindergarten  2.□ Primary/secondary school (grade____ )  3.□ Not attending school  4.□ Others_________________________ | | | |
| 7. Date of HIV diagnosis (dd/mm/yyyy) | □ no record | | | |
| 8. ART start date (dd/mm/yyyy) | □ no record | | | |
| 9. WHO clinical staging | □ no record | | | |
| 10. Initial CD4 test date (dd/mm/yyyy) | □ no record | | | |
| 11. CD4 count (absolute count & %) | _________(______%) □ no record | | | |
| 12. Initial Hb test date (dd/mm/yyyy) | □ no record | | | |
| 13. Hb level | □ no record | | | |
| 14. ART regimen  (name of ARVs taken by child) |  | | | |
| 15. Child knows HIV status *(according to the patients file)* | 1.□ Yes 2.□ No 3.□ Unknown  4.□ Not recorded | | | |
| 16. Referred from  *(if not recorded in the file, ask the caregiver)* | 1.□ PMTCT  2.□ OPD  3.□ IPD  4.□ General VCT  5.□ Others________________________ | | | |
| 17. PMTCT medicine taken by mother  *(mark all that apply)*  *(if not recorded in the file, ask the caregiver)* | 1.□ NVP  2.□ AZT (ZDV)  3.□ Others__________________  4.□ Nothing → go to 19  5.□ Unknown → go to 19 | | | |
| 18. PMTCT medicine taken during  *(mark all that apply)*  *(if not recorded in the file, ask the caregiver)* | 1.□ antenatal  2.□ intrapartum  3.□ postnatal | | | |
| 19. PMTCT medicine taken by child  *(mark all that apply)*  *(if not recorded in the file, ask the caregiver)* | 1.□ NVP  2.□ AZT (ZDV)  3.□ Nothing  4.□ Unknown | | | |
| 20. Breastfeeding  *(if not recorded in the file, ask the caregiver)* | 1.□ Breastfed until ______year______months  2.□ Never breastfed  3.□ Unknown | | | |
| 1. **Information of the Caregiver** | | | | |
| 21. Name of caregiver |  | | | |
| 22. Relationship to child |  | | | |
| 23. Sex of caregiver | 1.□ Male 2.□ Female | | | |
| 24. Age of caregiver | Years old | | | |
| 25. Educational attainment of caregiver | 1.□ _________grade completed  2.□ Never attended school  3.□ Others__________________________  4.□ Declined to answer | | | |
| 26. Language spoken by caregiver |  | | | |
| 27. Marital status | 1.□ Never married  2.□ Married or cohabiting  3.□ Divorced or separated  4.□ Widowed  5.□ Declined to answer | | | |
| 28. What kind of work do you do?  *(unnecessary to read all items of 1-12. Check all items that clients raised as their job)* | 1.□ Agriculture  2.□ Fishing  3.□ Commerce/business  4.□ Clerical/employee  5.□ Professional  6.□ Skilled/semi-skilled labour  7.□ Unskilled labour  8.□ Student  9.□ Apprentice  10.□ Housewife/homemaker  11.□ None (No agriculture or housework?)  12.□ Other, please specify:_______________ | | | |
| 29. Contact number (mobile phone) |  | | | |
| 1. **Information of the Family** | | | | |
| 30. Main source of household income |  | | | |
| 31. Household income level per months | 1.□ < 50,000 Kwacha  2.□ 50,000 – 99,999  3.□ 100,000-199,999  4.□ 200,000-499,999  5.□ > 500,000 | | | |
| 32. Household monthly expenditure | 1.□ < 50,000 Kwacha  2.□ 50,000 – 99,999  3.□ 100,000-199,999  4.□ 200,000-499,999  5.□ > 500,000 | | | |
| 33. The child’s father | 1.□ Alive  2.□ Died  3.□ Unknown | | | |
| 34. HIV status of father | 1.□ Reactive  2.□ Non reactive  3.□ Unknown  4.□ Declined to answer | | | |
| 35. ART for father | 1.□ Is/was on ART  2.□ Not on ART  3.□ Unknown  4.□ Declined to answer | | | |
| 36. The child’s mother | 1.□ Alive  2.□ Died  3.□ Unknown | | | |
| 37. HIV status of mother | 1.□ Reactive  2.□ Non reactive  3.□ Unknown  4.□ Declined to answer | | | |
| 38. ART for mother | 1.□ Is/was on ART  2.□ Not on ART  3.□ Unknown  4.□ Declined to answer | | | |
| 39. Family member (with whom the child currently live with)  *including the caregiver | Relationship to the child | Age | HIV status  (reactive  /non reactive  /unknown) | On ART  (yes/no/  unknown) |
|  |  |  |  |  |
|  |  |  |  |  |
|  |  |  |  |  |
|  |  |  |  |  |
|  |  |  |  |  |
|  |  |  |  |  |
|  |  |  |  |  |
|  |  |  |  |  |
|  |  |  |  |  |
| 40. Usually how do you come to the hospital? | 1.□ On foot  2.□ Bicycle  3.□ Scooter/motorcycle  4.□ Mini Bus  5.□ Vehicle/Car  6.□ Ban  7.□ Others________________ | | | |
| 41. Distance from hospital (one-way - by means mentioned above) | 1.□ Within 1 hours  2.□ 1-2 hours  3.□ 2-3 hours  4.□ 3-6 hours  5.□ More than 6 hours | | | |
| 42. How much does it cost for coming here? (One-way) | ZMK | | | |

1. **Care and Treatment for the Child**
2. How was the child’s HIV status discovered?
3. Who knows the child’s HIV status in the family (including caregiver)?
4. Who is the main caregiver of the child?
5. Do you know the name of drugs which the child is taking?

1.□ Yes 2.□ No

What are they?

1. Do you know how long the child needs to take these drugs?

1.□ Yes 2. □ No

How long?

1. What are the important things you should remember when giving these drugs to the child?
2. How does the child take her/his medication?

1.□ By her/himself

2.□ Caregiver (specify who) gives the medication

3.□ Others (specify)

1. Does the child take medicine regularly? Any difficulties?
2. How many times has the child missed medication during the last 3 days?
3. How much of medication did the child took in the past month?

*It is not likely that most people would take all of their doses. A mark at the left end where there is a number zero means the child taken no medications. A mark in the middle means the child has taken about half of medications. A mark on the right end where you can see the number ten means the child have taken every single dose of medications. Please put a mark on this line somewhere between zero and ten to describe your best guess about how much of medication the child took in the previous month.*

| never took | | missed | | | | | | | | missed half | | missed | | | | | | | | never missed | |
| --- | --- | --- | --- | --- | --- | --- | --- | --- | --- | --- | --- | --- | --- | --- | --- | --- | --- | --- | --- | --- | --- |
|  |  | 54times | | 48times | | 42times | | 36times | |  |  | 24times | | 18times | | 12 times | | 6times | |  |  |
|  |  |  |  |  |  |  |  |  |  |  |  |  |  |  |  |  |  |  |  |  |  |
|  |  |  |  |  |  |  |  |  |  |  |  |  |  |  |  |  |  |  |  |  |  |

1. Have the child ever experienced drug side effects and/or any diseases/new conditions?

1.□ Yes

2.□ No

3.□ Don’t know

If yes, what are they?

1. How do you think about the current health condition of the child?
2. How do you think about the growth of the child?
3. Do you think should the child be informed about her/his status?

1.□ Yes

2.□ No

3.□ Don’t know

Why?

1. Does the child know her/his HIV status? If so, how was the child informed?

1.□ Yes

2.□ No → go to 16

3.□ Don’t know→ go to 16

If yes,

When?

By whom?

How?

How did the child react when s/he was informed about the disease?

→ go to 19

1. If the child is not informed about the status yet, how has the child told/explained about her/his disease (how does s/he understand about the disease)?
2. Are you planning to inform about her/his disease to the child?

1.□ Yes

2.□ No → go to 18

3.□ Don’t know → go to 19

If yes,

When?

Because…

By whom?

Because….

How?

Because…

1. Why are you not planning to tell? *(only for caregiver who answered ‘not going to tell the child’)*
2. Do you face any challenges regarding the child’s care and treatment? If so, what are they?
3. Has the child discriminated against in the family, community, and/or at school? If so, how?
4. Do you discuss with the child openly about the issues related to the health of the child?

(do not discuss at all - 0, discuss every issue openly - 10)

| 0 | |  |  |  | 5 | |  |  |  | 10 | |
| --- | --- | --- | --- | --- | --- | --- | --- | --- | --- | --- | --- |
|  |  |  |  |  |  |  |  |  |  |  |  |
|  |  |  |  |  |  |  |  |  |  |  |  |

Any comments

1. Does the child discuss with you openly about the issues related to her/his health?

(does not discuss at all - 0, discuss every issue openly - 10)

| 0 | |  |  |  | 5 | |  |  |  | 10 | |
| --- | --- | --- | --- | --- | --- | --- | --- | --- | --- | --- | --- |
|  |  |  |  |  |  |  |  |  |  |  |  |
|  |  |  |  |  |  |  |  |  |  |  |  |

Any comments

1. Do you have any concern for the child (not only about disease but in general)? If yes, what are they?
2. Do you receive any support (e.g. social, financial, etc.) for the care of the child? If so what are they?
3. Does the child participate in any self-supporting group activities? If so what are they?
4. What kinds of support would you like to have for the child?

| 0 | |  |  |  | 5 | |  |  |  | 10 | |
| --- | --- | --- | --- | --- | --- | --- | --- | --- | --- | --- | --- |
|  |  |  |  |  |  |  |  |  |  |  |  |
|  |  |  |  |  |  |  |  |  |  |  |  |

1. Are you satisfied with the services provided at the hospital (not satisfied - 0, totally satisfied - 10)? If yes, could you give us some examples? If no, could you give us the reasons?
2. What would you recommend to further improve the services at this hospital?

Thank you very much!
