# Supplementary material for: Disclosure to HIV-seropositive children in rural Zambia
Source: BMC Pediatr. 2018 Aug 18;18:272. doi: 10.1186/s12887-018-1252-2 (PMC6098826; doi:10.1186/s12887-018-1252-2)
Supplement: Supplementary file 2 — Interview guide for chidren. (DOCX 22 kb) [file 12887_2018_1252_MOESM2_ESM.docx]

**Additional file 2**

**Interview guide for chidren**

Questionnaire for children

Date of interview (dd/mm/yyyy)

The child’s ART number

The name of the child

Age of the child

Sex of the child Boy / Girl

Questions with underlines are only for children who know their status.

Drawing – healthy child and unhealthy child

1. What are the differences between healthy child and unhealthy child?
2. Do you feel more like this child here (healthy child) or more like that one (unhealthy child?

□ 1. Healthy child

□ 2. Unhealthy child

Why?

1. How are you feeling (i.e. health condition) today?
2. What have you eaten yesterday? (i.e. enough food at home?)
3. Can you tell me why you came to this clinic today? (i.e. reason of the visit)
4. Who usually brings you to the clinic?
5. Who usually helps you to take your medication?
6. Why are you taking these medications? (the drugs which you are taking are for?)
7. How do you take your medication?

□ 1. all by myself

□ 2. caregiver gives me

□ 3. don’t know

□ 4. others____________________________________

1. When do you take your medication?

□ 1. at _____________hours and ________________hours

□ 2. when caregiver gives me

□ 3. anytime

□ 4. don’t know

□ 5. others______________________________________

1. How many times did you miss medication during the last 3 days?
2. How much of medication did you take during the past month?

| 0 | |  |  |  | 5 | |  |  |  | 10 | |
| --- | --- | --- | --- | --- | --- | --- | --- | --- | --- | --- | --- |
|  |  |  |  |  |  |  |  |  |  |  |  |
|  |  |  |  |  |  |  |  |  |  |  |  |

(didn’t take at all – 0, took about half of them – 5, took them all – 10)

1. Is it easy or difficult for you to take your medication? Why?
2. (if the child knows the status already) How did you know about your disease?
3. ( if the child knows the status already) How did you feel when you learn about your status?

Because…

1. What do you think/feel about your disease?
2. Do you feel different from other children? If so, how?
3. When you have a problem, with whom do you discuss the problem?
4. Do you discuss everything with (primary caregiver)?

(do not discuss at all - 0, I tell and discuss everything with her/him - 10)

| 0 | |  |  |  | 5 | |  |  |  | 10 | |
| --- | --- | --- | --- | --- | --- | --- | --- | --- | --- | --- | --- |
|  |  |  |  |  |  |  |  |  |  |  |  |
|  |  |  |  |  |  |  |  |  |  |  |  |

1. Currently do you have any problems or concerns/worries?

1.□ Yes

2.□ No

3.□ Others______________________

If yes, what are they?

1. Are your friends nice to you?

1.□ Yes → go to 21.

2.□ No, some are not nice to me → go to 20.

3.□ Don’t know → go to 21.

1. How are they NOT nice to you? For example?

Why they are not nice to you?

1. What do you think about the staff at this hospital?
2. Is there anything which you would like to suggest to the hospital?

Thank you very much!
